# From chronic hidradenitis suppurativa to epidermoid carcinoma, from inflammation to cellular degeneration

**DOI:** 10.1093/jscr/rjaf935

**Published:** 2025-11-25

**Authors:** Saúl Sánchez Iglesias, Cristina de la Cruz Cuadrado, Julián de Pedro Conal

**Affiliations:** Hospital Universitario de Toledo, Avenida del Río Guadiana, Toledo 45007, Spain; Hospital Universitario de Toledo, Avenida del Río Guadiana, Toledo 45007, Spain; Hospital Universitario de Toledo, Avenida del Río Guadiana, Toledo 45007, Spain

**Keywords:** case report, hidradenitis suppurativa, squamous cell carcinoma

## Abstract

Hidradenitis suppurativa is a chronic inflammatory skin disease characterized by recurrent nodules, abscesses, and sinus tracts, most often located in axillary, inguinal, and gluteal regions. Malignant transformation into squamous cell carcinoma is a rare but serious complication. We present the case of a 50-year-old man with ˃10 years of poorly controlled hidradenitis suppurativa who developed painful gluteal discharge and multiple exophytic lesions. Imaging demonstrated large perianal and perineal masses with destruction of the coccyx, but no distant spread. Biopsy confirmed well-differentiated squamous cell carcinoma. Because of the local extent, the multidisciplinary team recommended radiotherapy as the primary treatment. Malignant degeneration of hidradenitis suppurativa is more frequent in long-standing, advanced disease, particularly in gluteal and perineal areas, and is associated with delayed diagnosis and high mortality. Early biopsy of suspicious lesions and multidisciplinary management are essential to improve prognosis.

## Introduction

Hidradenitis suppurativa is a chronic skin disease caused by inflammation of the hair follicles, most frequently affecting the axillae, buttocks, groin, and breasts. It manifests as painful nodules, abscesses, and sinus tracts. The most severe complication of this condition is malignant degeneration into squamous cell carcinoma, and, exceptionally, the well-differentiated verrucous carcinoma variant has been described.

## Case report

We report the case of a 50-year-old male with a diagnosis of chronic hidradenitis –suppurativa for over 10 years, poorly controlled. He presented to the emergency department with gluteal pain and discharge. Physical examination revealed multiple draining sinus tracts with purulent material and large exophytic tumors.

**Figure 1:**
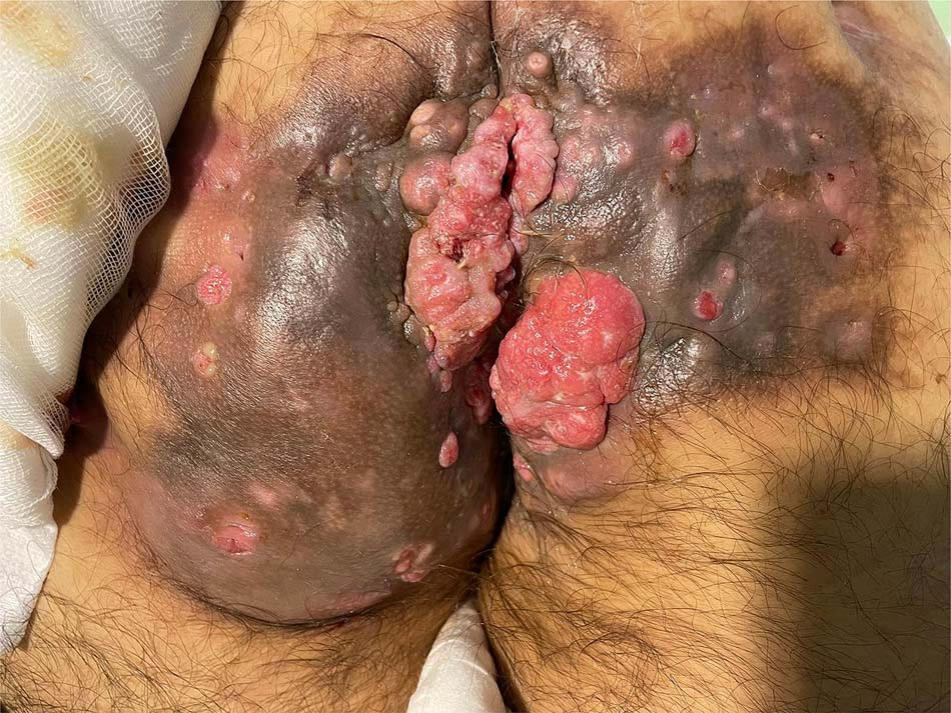
Massive squamous cell carcinoma.

He was admitted for antibiotic treatment, and further studies including thoracoabdominopelvic computed tomography (CT) and pelvic magnetic resonance imaging (MRI) were performed. Both revealed a large perianal and perineal mass overlying stage III hidradenitis suppurativa, with coccygeal bone destruction but no distant spread. Biopsies confirmed well-differentiated squamous cell carcinoma (Fig. 1). The case was presented to a multidisciplinary tumor board, which recommended radiotherapy due to the extent of locoregional disease.

## Discussion

Hidradenitis suppurativa is a chronic inflammatory condition that, although rare, can progress to squamous cell carcinoma [1, 2]. Malignant transformation is most frequently seen in perineal and gluteal sites, particularly in patients with long-standing disease and Hurley stage III [2, 3]. Reported incidence of squamous cell carcinoma in hidradenitis suppurativa ranges from 1% to 4.6%, though higher rates have been observed in selected series [1, 2]. Malignant transformation is typically associated with disease duration spanning 10 to over 40 years [3].

Mortality related to this complication is significant, reaching 40%–43% in some series, reflecting both the aggressiveness of the neoplasm and frequent late diagnosis [4]. Unusual histological variants, such as verrucous carcinoma, have been reported, mainly in male patients with chronic hidradenitis suppurativa [5, 6].

Pathophysiology is linked to ‘scar cancerization,’ driven by chronic inflammation, persistent abscesses, sinus tracts, and repeated skin injury. Risk factors such as smoking and immunosuppression, including anti-tumor necrosis factor (TNF) biologics, may promote malignant change [3]. Additionally, Human Papilloma Virus (HPV)-16 infection has been identified in some squamous cell carcinoma cases, suggesting a potential preventive role for HPV vaccination in high-risk patients [7].

Radical surgery remains the gold standard of treatment [2]. However, alternative options have been explored in selected cases. Cemiplimab, a PD-1 inhibitor approved for advanced cutaneous squamous cell carcinoma, has demonstrated efficacy in patients not suitable for surgery or curative radiotherapy [8].

Chemoradiotherapy may also be considered in order to preserve critical structures such as the anal sphincter, while surgery is reserved for extensive or refractory tumors [8, 9].

Given the severity of this complication, close monitoring of patients with chronic hidradenitis suppurativa is essential, especially those with perineal or gluteal involvement, long-standing disease, or advanced stages [2, 3]. Biopsy of exophytic, non-healing, or rapidly growing lesions facilitates early diagnosis [2]. A multidisciplinary approach—including surgery, dermatology, oncology, and radiology—is crucial to optimize evaluation and therapeutic decisions [9].

Emerging evidence also supports the potential role of HPV vaccination as a preventive strategy, though further studies are needed to validate its effectiveness [7].

## Conclusions

Malignant degeneration of chronic hidradenitis suppurativa into squamous cell carcinoma is an uncommon but serious complication, resulting from the synergistic effects of chronic inflammation, impaired cellular immunity, and, in some cases, HPV infection. The well-differentiated variant exhibits high potential for local infiltration with a low rate of distant metastasis. Treatment may require extensive surgery or chemoradiotherapy depending on disease stage. Prognosis is often poor due to tumor extent or metastasis at diagnosis.
